# IRSp53 accumulates at the postsynaptic density under excitatory conditions

**DOI:** 10.1371/journal.pone.0190250

**Published:** 2017-12-28

**Authors:** Ayse Dosemeci, Amelia Burch, Hannah Loo, Dana Toy, Jung-Hwa Tao-Cheng

**Affiliations:** 1 Laboratory of Neurobiology, National Institute of Neurological Disorders and Stroke, National Institutes of Health, Bethesda, Maryland, United States of America; 2 EM Facility, National Institute of Neurological Disorders and Stroke, National Institutes of Health, Bethesda, Maryland, United States of America; Bilkent University, TURKEY

## Abstract

IRSp53 (BAIAP2) is an abundant protein at the postsynaptic density (PSD) that binds to major PSD scaffolds, PSD-95 and Shanks, as well as to F-actin. The distribution of IRSp53 at the PSD in cultured hippocampal neurons was examined under basal and excitatory conditions by immuno-electron microscopy. Under basal conditions, label for IRSp53 is concentrated at the PSD. Upon depolarization by application of a medium containing 90 mM K^+^, the intensity of IRSp53 label at the PSD increased by 36±7%. Application of NMDA (50 μM) yielded 53±1% increase in the intensity of IRSp53 label at the PSD compared to controls treated with APV, an NMDA antagonist. The accumulation of IRSp53 label upon application of high K^+^ or NMDA was prominent at the deeper region of the PSD (the PSD pallium, lying 40–120 nm from the postsynaptic plasma membrane). IRSp53 molecules that accumulate at the distal region of the PSD pallium under excitatory conditions are too far from the plasma membrane to fulfill the generally recognized role of the protein as an effector of membrane-bound small GTPases. Instead, these IRSp53 molecules may have a structural role organizing the Shank scaffold and/or linking the PSD to the actin cytoskeleton.

## Introduction

IRSp53 (Insulin receptor substrate p53, also called BAIAP2), a protein expressed in several cell types, is implicated in the regulation of membrane curvature and actin cytoskeleton (review: [1]). IRSp53 contains several protein-protein interaction domains. The N-terminal IMD (I-BAR) domain of IRSp53 can bind to membranes and also promotes self-association to produce head-to-head dimers with a C-terminal protruding on each end. The C-terminal contains a CRIB-PR domain that interacts with the small GTPase Cdc42, and SH3 domain that interacts with a number of actin-regulating proteins, including Eps8, Espin, Mena, N-WASP and WAVE (reviews: [2], [3]). By virtue of its capacity to regulate membrane and actin dynamics, IRSp53 is involved in formation of filopodia and lamellipodia in various cell types [4], [5], [6], (review: [3]).

IRSp53 is also expressed in neurons and appears to be involved in several nervous system-specific processes. Downregulation of the protein in cultured neurons causes a decrease in the density and size of dendritic spines [7]. Knock out or reduction of IRSp53 results in increased levels of NMDA receptors at the PSD and enhanced long-term potentiation in the hippocampus as well as severe learning deficits [8] [9]. In humans, the gene has been linked to various neurological disorders including autism [10], ADHD [11] and schizophrenia [12], [13].

IRSp53 in neurons is especially concentrated at the postsynaptic density (PSD), a protein complex lining the postsynaptic membrane comprising a 30–40 nm thick electron-dense core layer and a deeper, contiguous pallium layer [14]. Immuno-electron microscopy shows dense labeling for IRSp53 mainly at the PSD core [15]. PSD fractions from brain tissue show enrichment in IRSp53 [16] and quantitative mass spectrometric analysis of isolated PSDs from cerebral cortex yields a stoichiometric ratio of roughly one IRSp53 to four PSD-95 [17]. IRSp53 associates with BAI1, an adhesion G protein-coupled receptor (review: [18]), PSD-95 [19], the major scaffold protein at the PSD core, and Shanks [20], the major scaffold proteins at the PSD pallium, through distinct domains.

Although IRSp53 is known to be a major protein at the PSD whose deficiency causes major functional defects, the precise role of the protein in the organization and function of the PSD is not clear. In previous studies, examination of the movement of proteins at and around the PSD during activity, including translocation of CaMKII to the PSD, provided insights into molecular mechanisms underlying synaptic function and plasticity (review [14]). In a similar vein, in the present study, we set out to examine changes in levels and localization of synaptic IRSp53 under excitatory conditions.

## Materials and methods

The animal protocol was approved by the National Institute of Neurological Disorders and Stroke/ National Institute of Deafness and Communication Disorders/National Center for Complementary and Integrative Health Animal Use and Care Committee and conforms to NIH guidelines. Approval #Asp 1159–15.

### Antibodies

Rabbit polyclonal PSD-95 antibody was custom produced by New England Peptide (Gardner, MA; 1:5000 for Western). Mouse monoclonal panShank antibody (Clone N23B/49) was obtained from Neuromab (Davis, CA; 1:1000 for Western). Two antibodies were used for detection of IRSp53. Antibody 1 was a mouse monoclonal antibody (Clone L117/1) raised against a peptide corresponding to AAs 1–250 of human IRSp53 from Neuromab (1:4 for Western, undiluted for immunoEM). Antibody 2 was a rabbit polyclonal antibody raised against a peptide corresponding to AAs 1–300 of human IRSp53 from Protein Tech Group (Rosemont, IL; 1:1000 for Western, 1:150–250 for immunoEM).

### Preparation of subcellular fractions from rat brain and Western immunoblotting

Brains from adult Sprague Dawley rats were supplied by Rockland Immunochemicals Inc. (Gilbertsville, PA). Animals were subjected to 1 min CO_2_ prior to decapitation. Brains were collected within two minutes of decapitation and were immediately frozen in liquid nitrogen. Brains were rapidly thawed in isotonic sucrose solution and dissected to remove white matter.

Cerebral cortices were homogenized in isotonic sucrose. Synaptosome and PSD fractions were prepared essentially as described previously [21]. Proteins in homogenate, synaptosome, and PSD fractions were resolved by SDS-PAGE using 4–15% Mini-PROTEAN TGX Precast polyacrylamide gels (BioRad). Gels were transferred to PVDF membranes using the Trans-Blot Turbo Transfer System (BioRad), blocked, incubated with primary and secondary antibodies, and visualized via chemiluminescence (BioRad).

### Preparation and treatment of hippocampal cultures

All animals were housed in an NIH intramural research program vivarium as described before [22]. Ten Sprague Dawley timed pregnant rats from Taconic Farms (Germantown, MD, USA) and Charles River (Raleigh, NC, USA) were used. Pregnant dams were euthanized by CO_2_ inhalation. Embryos were then collected by caesarian section and decapitated with sharp scissors (Animal protocol Number:ASP1159).

Cell cultures were prepared as previously described [22]. Briefly, hippocampi from embryonic 20 days-old rat fetuses were dissociated by papain, and then plated onto previously prepared rat glial feeder cultures. Cultures were maintained in MEM Eagle Salts and kept in 10% CO_2_ incubator at 35°C, and experiments were carried out with three weeks-old cultures.

Culture dishes were placed on a floating platform in a water bath maintained at 37°C. Control incubation medium was: 124 mM NaCl, 2 mM KCl, 1.24 mM KH_2_PO_4_, 1.3 mM MgCl_2_, 2.5 mM CaCl_2_, 30 mM glucose in 25 mM HEPES at pH 7.4. High K^+^ medium was at 90 mM KCl, with osmolarity compensated by reducing the concentration of NaCl. NMDA was added to control medium at 50 μM concentration.

For high K^+^ experiments, cell cultures were washed with control medium and treated for 30 seconds with either control or high K^+^ media, and then fixed immediately. The timing of treatment at 30 s was chosen because this short treatment yielded consistent results while a longer treatment at 2 min yielded highly variable results. We attribute the high variability in labeling intensity of IRSp53 following longer excitatory stimuli to changes in affinity for the antibody upon phosphorylation. Indeed, phosphorylation of IRSp53 in isolated PSD fractions resulted in a marked decrease in antibody recognition in Western immunoblots (S1 Fig).

For NMDA experiments, control samples were pre-incubated for 5 min in medium containing 50 μM APV, an NMDA receptor antagonist, followed by 30 s mock-treatment in the same medium. In parallel, the designated ‘NMDA treatment’ samples were pre-incubated in control medium for 5 min, followed by a 30 s treatment in medium containing 50 μM NMDA.

### Pre-embedding immunogold labelling and electron microscopy

All steps were carried out at room temperature unless otherwise indicated. Cell cultures were fixed with 2% paraformaldehyde (EMS, Fort Washington, PA) in PBS for 15–20 min, washed and stored in PBS at 4˚C until ready for immunolabeling. Cells were permeablized and blocked with 0.1% saponin and 5% normal goat serum in PBS for 30 min, incubated with primary antibodies for 1 hr, washed and incubated with secondary antibodies (Nanogold from Nanoprobes, Yaphand, NY) for 1 hr, washed and fixed with 2% glutaraldehyde in PBS, stored in fixative at 4˚C until ready for silver enhancement. Samples were washed in deionized water, then silver enhanced (HQ kit from Nanoprobes), treated with 0.2% osmium tetroxide in 0.1 M phosphate buffer at pH 7.4 on ice for 30 min, followed by treatment with 0.25% uranyl acetate in 0.1 N acetate buffer at pH 5.0 on ice for 30 min, then dehydrated through a graded series of ethanols and finally embedded in epoxy resins.

Thin sections of 70–90 nm were cut *en face* and picked up on 400 mesh grids, and counter-stained with uranyl acetate and lead citrate. Images were taken with a digital CCD camera (AMT XR-100, Danvers, MA, USA).

### Morphometry

**Fig 1:**
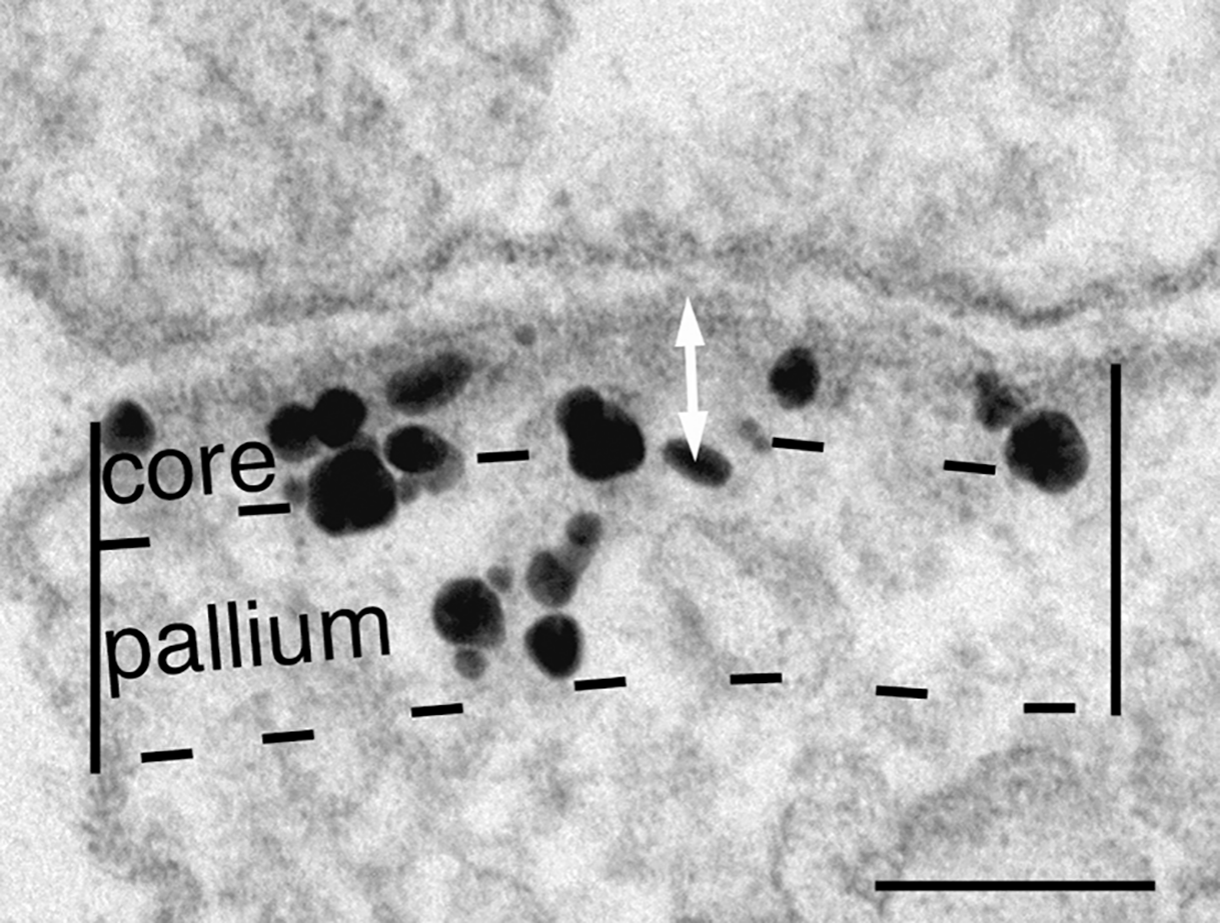
Electron micrograph of an asymmetric synapse in dissociated hippocampal cultures, labelled with an antibody (Ab1) for IRSp53. To assess the labeling intensity at the PSD, two parallel lines, 120 nm deep, were drawn from the edges of the postsynaptic density into the cytoplasm, then a dashed line was drawn parallel to the postsynaptic membrane to mark the area for measurement. This dashed line is curved consistent with the curvature of the plasma membrane. Gold particles (seen as black grains of heterogeneous size) within the area were counted and the number was divided by the length of the PSD to yield the labeling intensity of the PSD (# label/μm PSD). Distance from the postsynaptic membrane for each gold particle was measured from the center of the particle to the outer edge of the postsynaptic membrane (white arrow). This image was from a depolarized sample. Scale bar = 0.1 μm.

At least 4–5 grid openings were randomly chosen from each thin-sectioned sample and every cross-sectioned asymmetric synapse encountered was photographed for morphometry. The PSD region was arbitrarily marked as the area extending 120 nm beneath the postsynaptic membrane [23]. This area was further divided into two compartments–the proximal PSD core within 40 nm of the postsynaptic membrane, and the distal PSD pallium, 40–120 nm from the postsynaptic membrane (Fig 1; cf. [14]). Labeling intensity at the PSD is defined as number of gold particles within the 120 nm depth of the PSD, divided by the length of the PSD. Statistical significance of the differences in labeling intensity between groups was assessed by Student’s t test.

The distance of each gold particle within the PSD from the postsynaptic membrane was measured. For distance measurements, segments of clearly cross-sectioned postsynaptic membranes were marked and the distance of each gold particle underneath the segment was measured. All distance measurements from a sample were plotted into a histogram to illustrate the laminar distribution of label from the postsynaptic plasma membrane. Because the distribution is typically skewed, the significance of the differences in the median distances between groups were assessed by Wilcoxon rank-sum test (KaleidaGraph, Synergy Software, Reading, PA).

## Results

**Fig 2:**
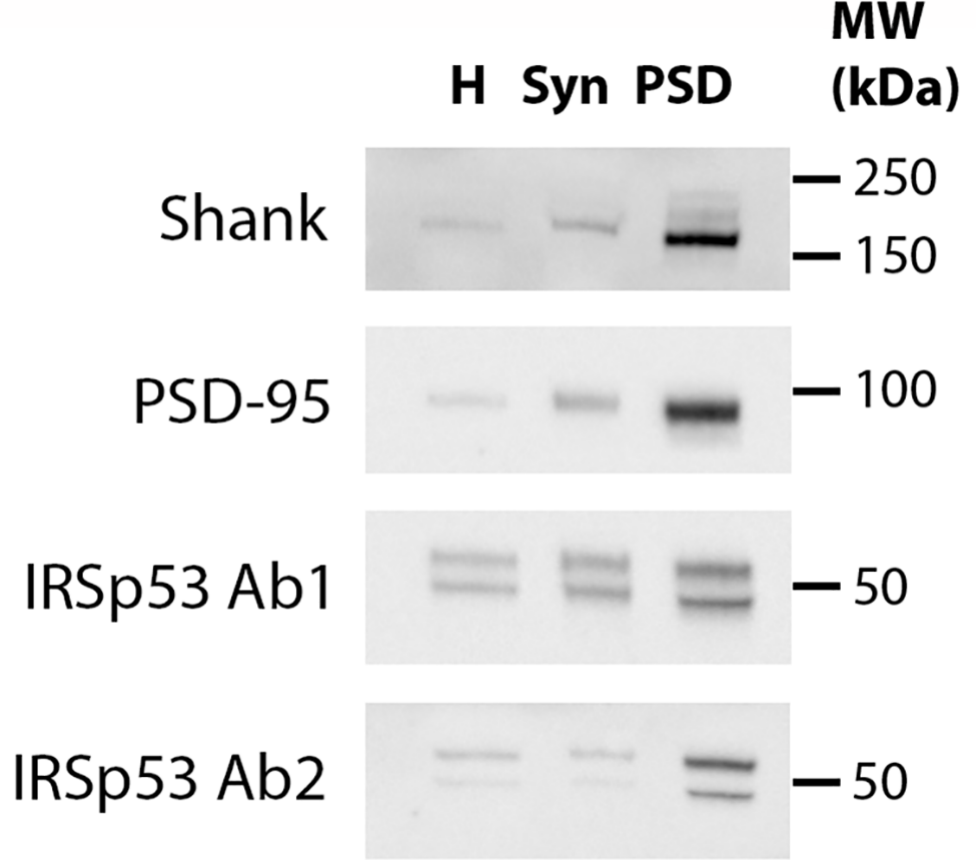
Western immunoblots with two antibodies (Ab1 and Ab2) for IRSp53 show enrichment of the protein in PSD fractions. Fractions were isolated from cerebral cortices of rat brains and probed for two PSD markers, Shank and PSD-95, as well as IRSp53 (10 μg protein/lane). Homogenate (H), Synaptosome (Syn), Postsynaptic density (PSD).

The subcellular distribution of IRSp53 was studied using biochemistry and electron microscopy with two different antibodies. In Western immunoblots from preparations from adult cerebral cortex, both antibodies recognized a doublet around 50 kDa (Fig 2). Comparison of subcellular fractions showed enrichment of IRSp53 in the PSD fraction compared to parent homogenate and synaptosome fractions, in parallel with two PSD markers, Shank and PSD-95 (Fig 2).

**Fig 3:**
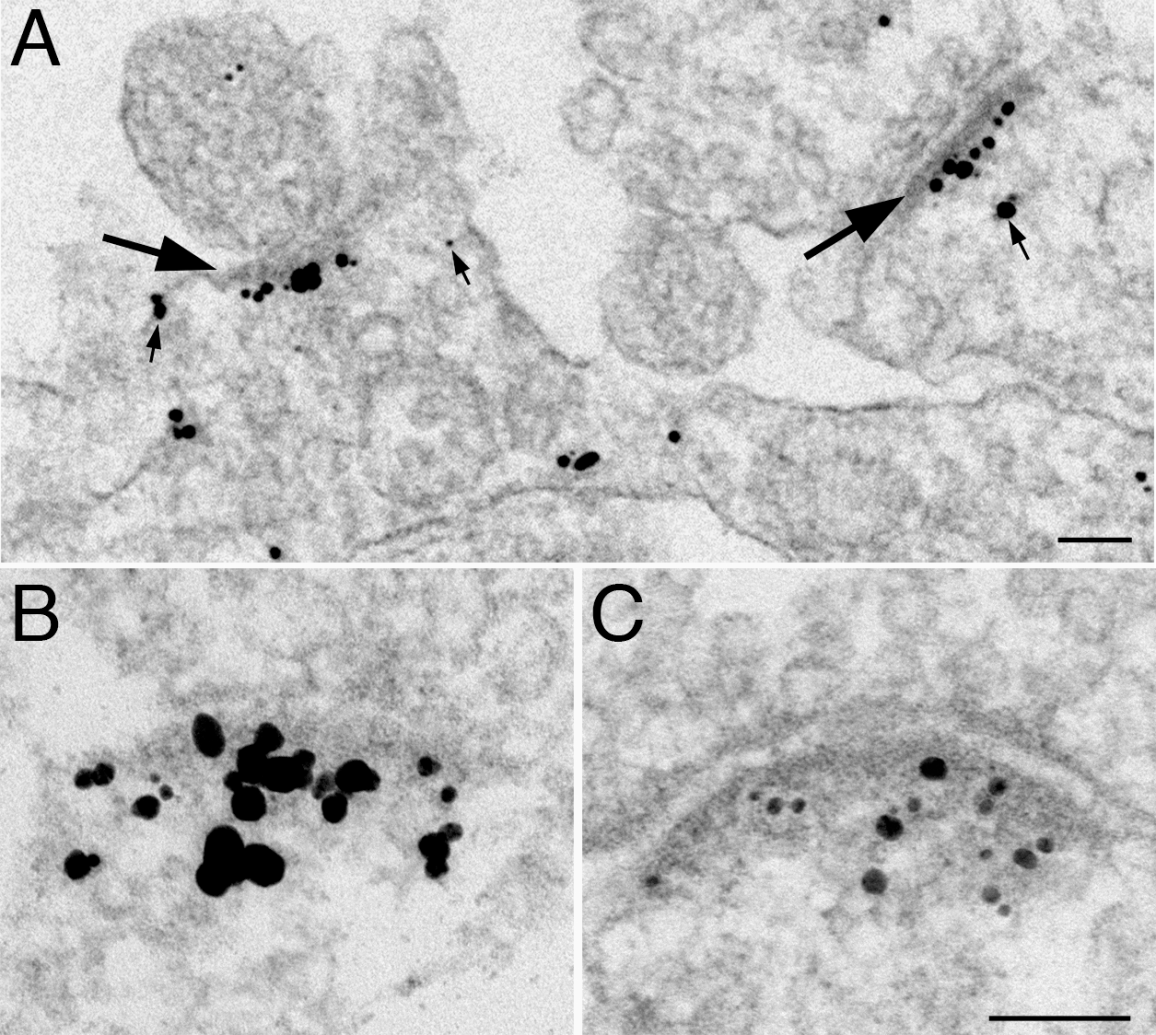
Excitatory stimuli promote increased labelling for IRSp53 at the PSD. Dissociated hippocampal neuronal cultures were labeled with IRSp53 antibodies (A, C with Ab1; B with Ab2) under basal conditions (A), following depolarization with high K^+^ (B), or treatment with NMDA (C). Under basal conditions, label is typically concentrated at the PSD (large arrows in A) with some scattered label in the cytoplasm (small arrows in A). Upon stimulation (B, C), more label accumulates on the PSD. Scale bar = 0.1 μm, B&C share the same bar.

In agreement with biochemical results demonstrating enrichment of IRSp53 in PSD fractions, pre-embedding immuno-electron microscopy using either IRSp53 antibody 1 (Fig 3A and 3C) or antibody 2 (Fig 3B) showed specific labeling of PSDs (region extending 120 nm from the postsynaptic membrane, including PSD core and PSD pallium). Brief depolarization by the application of high K^+^ (30 s in 90 mM K^+^) promoted a 36±7% increase in IRSp53 label intensity at the PSD in three experiments, each reaching statistical significance (Fig 3B, Table 1). In order to test possible involvement of NMDA receptors in mediating the effect of depolarization, in a second series of experiments, cultures were treated with either an NMDA receptor antagonist, APV, or with NMDA (30 s in 50 μM). PSDs in NMDA-treated cells exhibited 53±1% more IRSp53 label compared to APV-treated controls in three experiments, each reaching statistical significance (Fig 3C, Table 1), indicating that activation of NMDA receptors is sufficient to induce translocation of IRSp53 to the PSD.

**Table 1:** Labeling intensity for IRSp53 in the PSD under control and excitatory conditions.

		Labeling intensity (# label/μm PSD) mean ± SEM (n) = number of synaptic profiles measured		Percent increase (Student’s t-test)
		Control	High K^+^	
Exp 1	Antibody 1	24.1 ± 1.9 (52)	35.0 ± 2.7 (32)	45% (P<0.0001)
Exp 2		24.1 ± 2.6 (29)	34.0 ± 2.8 (41)	41% (P<0.05)
Exp 3	Antibody 2	34.5 ± 2.1 (73)	42.1 ± 2.0 (81)	22% (P<0.05)
		**APV**	**NMDA**	
Exp 4	Antibody 1	23.0 ± 1.8 (42)	35.7 ± 2.5 (30)	55% (P<0.0005)
Exp 5	Antibody 2	28.0 ± 2.2 (45)	42.6 ± 2.3 (49)	52% (P<0.0001)
Exp 6		17.5 ± 1.6 (37)	26.9 ± 2.3 (37)	53% (P<0.005)

Experiments with isolated PSDs indicate that CaMKII-mediated phosphorylation of IRSp53 results in a decrease in its affinity for both antibodies (S1 Fig). The sensitivity of the antibodies to the phosphorylation state of IRSp53 presents a caveat for the interpretation of results in experiments where a change in phosphorylation is expected. Indeed, NMDA treatment was shown to promote activation of CaMKII in cultured hippocampal neurons [24], [25]. Although, in the present study, the duration of excitatory treatments (high K^+^, NMDA) was limited to 30s, some degree of IRSp53 phosphorylation and decreased antibody signal intensity would still be expected even under these relatively short excitatory treatments. In this case the reported increases in IRSp53 label intensity would represent underestimates of the increases in actual IRSp53 protein levels. The alternative possibility of net dephosphorylation seems unlikely but cannot be excluded.

**Fig 4:**
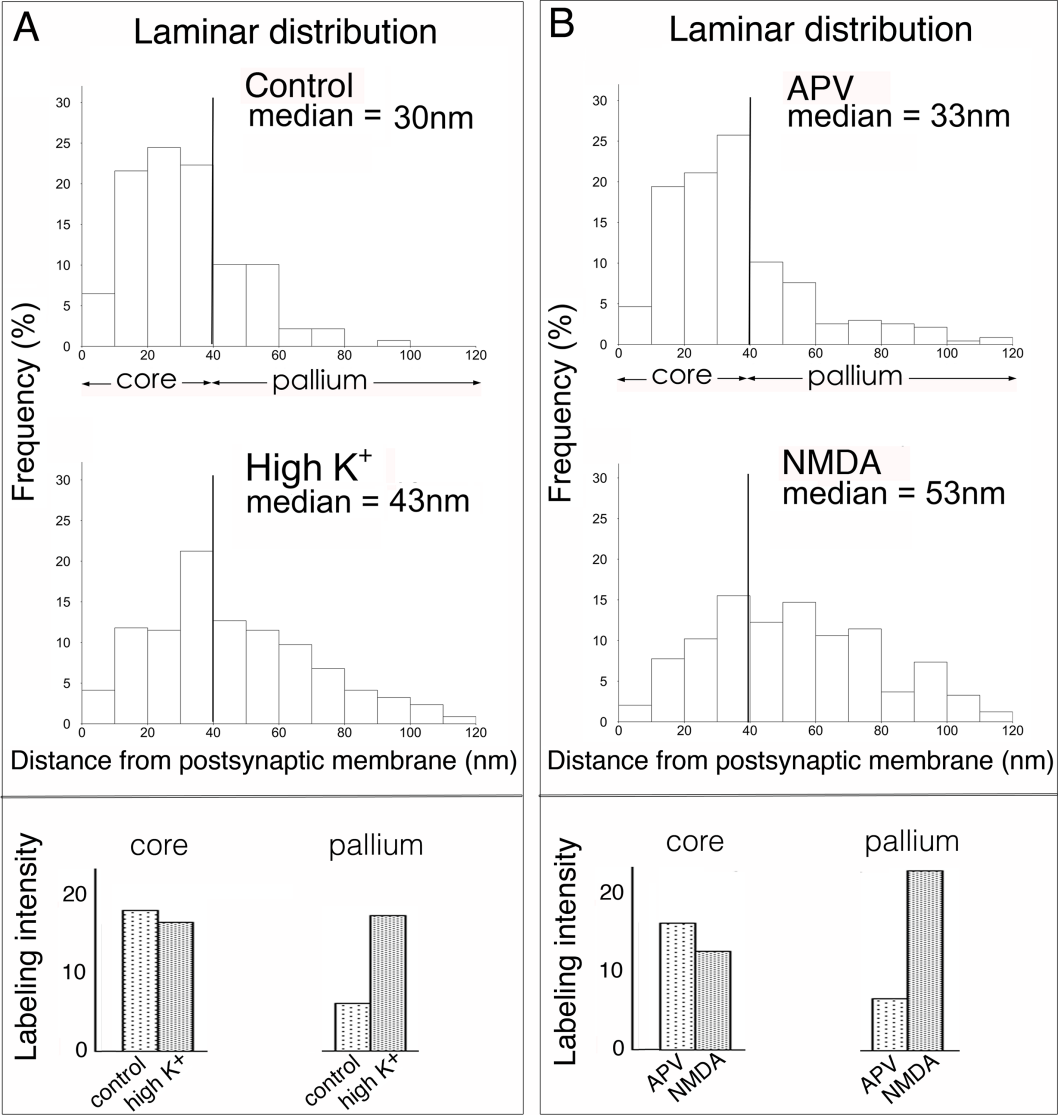
Treatment with high K^+^ (**A**) or NMDA (**B**) promotes accumulation of IRSp53 label at the PSD pallium. Data shown are from two representative experiments (see S2 and S3 Figs for all six experiments). Hippocampal cultures were incubated for 30 s in control medium or in media containing high K^+^ or NMDA. Distances of gold particles from the postsynaptic membrane were measured. **Top panels:** Histograms show the percentage of label located in consecutive laminar layers (10 nm bins). **Bottom panels:** Labeling intensities in core and pallium compartments were estimated as: labelling intensity in PSD x percentage of label in each compartment.

In order to assess the accumulation of IRSp53 label into different layers of the PSD, the distances of gold particles from the postsynaptic membrane were measured. Distance measurements allowed for the estimation of the relative amounts of label in the core and pallium compartments. In a typical experiment (Fig 4A), under control conditions, the label for IRSp53 was concentrated at the PSD core, with 75% of the total label located within this region. Following depolarization with K^+^, ~50% total label located within the PSD core and ~50% within the PSD pallium, indicating addition of label to the pallium. Treatment with NMDA produced similar results (Fig 4B). This trend was consistent in all six experiments using either antibody 1 or antibody 2 (S2 Fig). On average, the percentage of total label within the PSD core was 70.8 ± 4.4% under control conditions and 48.1 ± 8.3% under stimulated conditions (P<0.001, paired t test). The median distance from the membrane under control conditions or APV treatment was 27–33 nm. The median distance of gold particles shifted to 37–53 nm under excitatory conditions (S2 Fig), differences reaching statistical significance in all experiments. On average, the median distance of gold particles from the membrane shifted from 31.7 ± 1.1 nm under basal conditions to 43.3 ± 2.4 nm upon stimulation (means of median distances from 6 experiments, P<0.005, paired t test).

Data on mean labeling intensity for the entire PSD (Table 1) and percentages of label contained in the core and pallium allows estimation of the average labeling intensities in individual compartments (Fig 4A and 4B; lower panels). Comparison of labeling intensities from samples under basal and excitatory conditions showed a conspicuous increase in the labeling intensity at the PSD pallium but not at the PSD core (Fig 4A and 4B; lower panels). This trend was consistent in all six experiments (paired t test: P<0.0005, S3 Fig) with 1.7–2.5 fold increases upon treatment with high K^+^ and 2.6–3.4 fold increases upon treatment with NMDA in the labelling intensity at the PSD pallium but no appreciable changes at the PSD core.

## Discussion

The present study examined the effect of activity on the distribution of IRSp53 at the PSD. A previous study by fluorescence microscopy had shown that stimulation with NMDA causes translocation of the protein from the dendrites to spines [26]. Here, using immuno-electron microscopy, we demonstrate that depolarization, as well as NMDA application, promotes accumulation of IRSp53 at the PSD, with a prominent increase at the deeper pallial layer of the complex, 40–120 nm from the postsynaptic membrane.

Our observation that ~70% of IRSp53 label at the PSD is located within the PSD core (0–40 nm from the postsynaptic membrane) under basal conditions is consistent with a previous immuno-EM study on adult rat brain [15]. Following excitatory stimuli, the distribution of label becomes more widespread throughout the PSD due to prominent accumulation into the pallium (Fig 4). As mentioned above, IRSp53 has multiple potential binding partners, including PSD-95 and Shanks, major scaffolds at the PSD core and PSD pallium respectively. We presume that the anchoring of IRSp53 into different layers of the PSD is achieved through its binding to different sets of partners. Thus, it is likely that IRSp53 molecules within the PSD core would be anchored to PSD-95, with or without simultaneous binding to Shanks, whereas IRSp53 molecules within the distal region of the pallium may be solely anchored to Shanks.

Localization of IRSp53 at different layers of the PSD implies that it may fulfill different functional roles. Localization of IRSp53 close to the postsynaptic plasma membrane would be in line with its proposed function in mediating downstream signaling from the membrane-bound small GTPases, Rac and Cdc42 (review: [1]). In addition, IRSp53 molecules near the core-pallium interface may have a structural role as adaptors linking PSD-95 to Shanks [19], in a similar fashion to GKAPs.

IRSp53 molecules that accumulate into the distal region of the PSD pallium under excitatory conditions, however, are too far away from the plasma membrane to fulfill signaling functions linked to membrane-bound receptors and are not expected to interact with the membrane to regulate or maintain curvature. An adaptor function between PSD-95 and Shanks would also be excluded for IRSp53 molecules at the distal region of the PSD pallium. On the other hand, considering the Shank- and actin-binding capacity of IRSp53, these molecules at the deeper edge of the PSD pallium are optimally located to serve as adaptors between the PSD and the actin cytoskeleton.

Under excitatory conditions Shanks preferentially accumulate at the pallium [23]. At present, it is not clear whether the translocation of Shanks and of IRSp53 are coordinated events, nor how these newly accumulated molecules would be organized at the PSD pallium. A possible scenario is suggested for the involvement of IRSp53 in organizing the Shank scaffold: IRSp53 is known to self-associate to produce a dimer with two Shank-binding SH3 domains at each end [27]. Such dimers would have cross-linking capacity, anchoring themselves to Shank molecules already located at the PSD through one end, and tethering incoming Shank molecules through their other end.

In conclusion, immuno-electron microscopy allowed evaluation of the levels and distribution of IRSp53 within the PSD under basal and excitatory conditions at the ultrastructural level. These results reveal an activity-regulated pool of IRSp53 at the deeper region of the PSD that may be involved in the organization of the Shank scaffold as well as linking the scaffold to the actin cytoskeleton.

## Supporting information

S1 Fig CaMKII-mediated phosphorylation of IRSp53 decreases its affinity for both antibodies. Isolated PSD fractions were incubated under different conditions as indicated on top. Protocols for the preparation of PSD fraction and phosphorylation were as described in Dosemeci et al., 2016 (FEBS Lett., 590:2934–9). Figure shows Western immunoblots from those samples using antibody1 (Ab1) or antibody2 (Ab2). A change in electrophoretic mobility in the presence of ATP is indicative of phosphorylation. In the presence of ATP, Ca^2+^and calmodulin (Ca/CM), the change in mobility is most pronounced and a marked reduction in the affinity for both antibodies is observed. Ca^2^+/calmodulin-induced changes are blocked by CN21, a specific inhibitor for CaMKII, indicating that CaMKII activation promotes phosphorylation of IRSp53 at the PSD.
(DOCX)

S2 Fig Laminar distribution of IRSp53 label at the PSD under control/APV and excitatory conditions. Data for all six experiments is presented. Hippocampal cultures were exposed for 30 s to media containing high K^+^ or NMDA. Distances of gold particles from the postsynaptic membrane were measured. Histograms show the percentage of label located in consecutive layers (10 nm bins). The median distance of gold particles from the postsynaptic membrane showed a significant increase under excitatory conditions in all six experiments (P<0.0001, Wilcoxon test, n = number of gold particles measured).
(PDF)

S3 Fig Labelling intensity in the PSD core and PSD pallium under control/APV and excitatory conditions. Data from all six experiments is presented. Labeling intensities in core and pallium compartments were estimated as: labelling intensity in PSD x percentage of label in each compartment. There was a significant difference in label intensity between basal (control and APV-treated) and stimulated (high K^+^ and NMDA-treated) samples in the pallium (P<0.0005, paired t test) but not in the core.
(PDF)
