# Supplementary material for: IRSp53 accumulates at the postsynaptic density under excitatory conditions
Source: PLoS One. 2017 Dec 28;12(12):e0190250. doi: 10.1371/journal.pone.0190250 (PMC5746258; doi:10.1371/journal.pone.0190250)
Supplement: S1 Fig — Isolated PSD fractions were incubated under different conditions as indicated on top. Protocols for the preparation of PSD fraction and phosphorylation were as described in Dosemeci et al., 2016 (FEBS Lett., 590:2934–9). Figure shows Western immunoblots from those samples using antibody1 (Ab1) or antibody2 (Ab2). A change in electrophoretic mobility in the presence of ATP is indicative of phosphorylation. In the presence of ATP, Ca2+and calmodulin (Ca/CM), the change in mobility is most pronounced and a marked reduction in the affinity for both antibodies is observed. Ca2+/calmodulin-induced changes are blocked by CN21, a specific inhibitor for CaMKII, indicating that CaMKII activation promotes phosphorylation of IRSp53 at the PSD. (DOCX) [file pone.0190250.s001.docx]

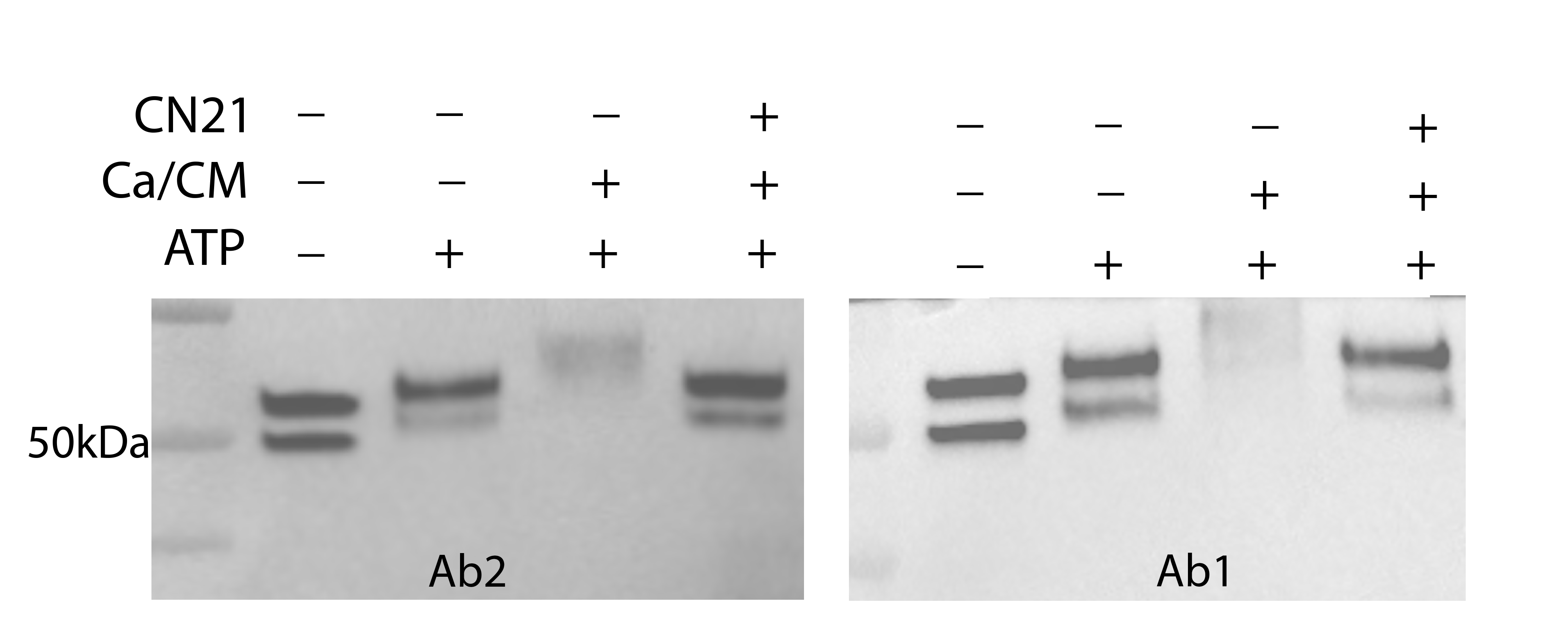


S1 Fig. CaMKII-mediated phosphorylation of IRSp53 decreases its affinity for both antibodies.

Isolated PSD fractions were incubated under different conditions as indicated on top. Protocols for the preparation of PSD fraction and phosphorylation were as described in Dosemeci et al., 2016 (FEBS Lett., 590:2934-9). Figure shows Western immunoblots from those samples using antibody1 (Ab1) or antibody2 (Ab2). A change in electrophoretic mobility in the presence of ATP is indicative of phosphorylation. In the presence of ATP, Ca^2+^and calmodulin (Ca/CM), the change in mobility is most pronounced and a marked reduction in the affinity for both antibodies is observed. Ca^2^+/calmodulin-induced changes are blocked by CN21, a specific inhibitor for CaMKII, indicating that CaMKII activation promotes phosphorylation of IRSp53 at the PSD.
