# Supplementary material for: IRSp53 accumulates at the postsynaptic density under excitatory conditions
Source: PLoS One. 2017 Dec 28;12(12):e0190250. doi: 10.1371/journal.pone.0190250 (PMC5746258; doi:10.1371/journal.pone.0190250)
Supplement: S2 Fig — Data for all six experiments is presented. Hippocampal cultures were exposed for 30 s to media containing high K+ or NMDA. Distances of gold particles from the postsynaptic membrane were measured. Histograms show the percentage of label located in consecutive layers (10 nm bins). The median distance of gold particles from the postsynaptic membrane showed a significant increase under excitatory conditions in all six experiments (P<0.0001, Wilcoxon test, n = number of gold particles measured). (PDF) [file pone.0190250.s002.pdf]

Control vs. High K^+

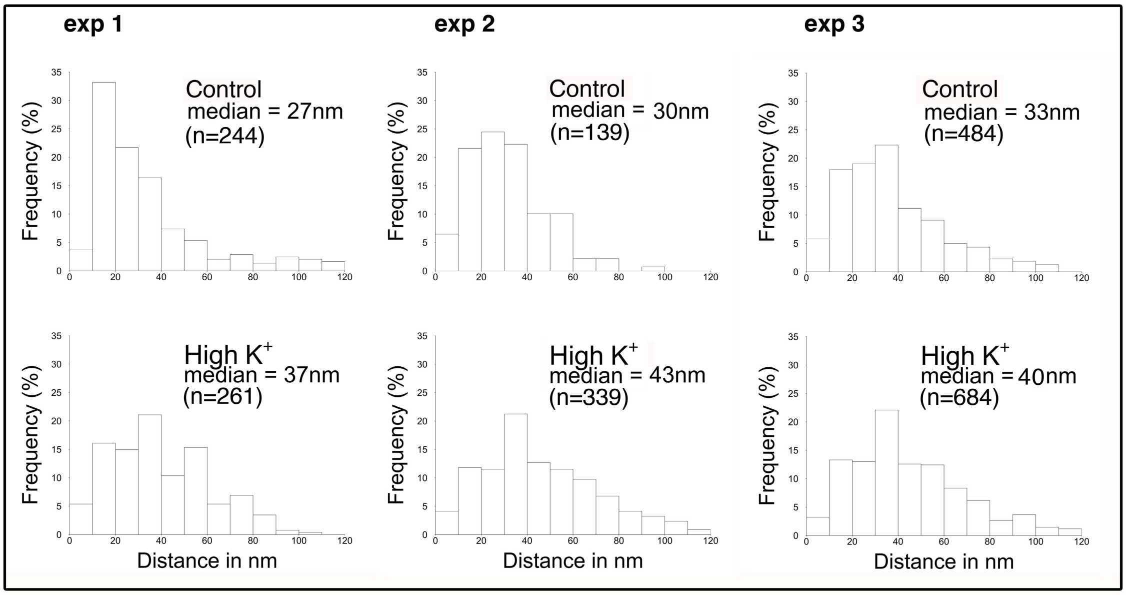

APV vs NMDA

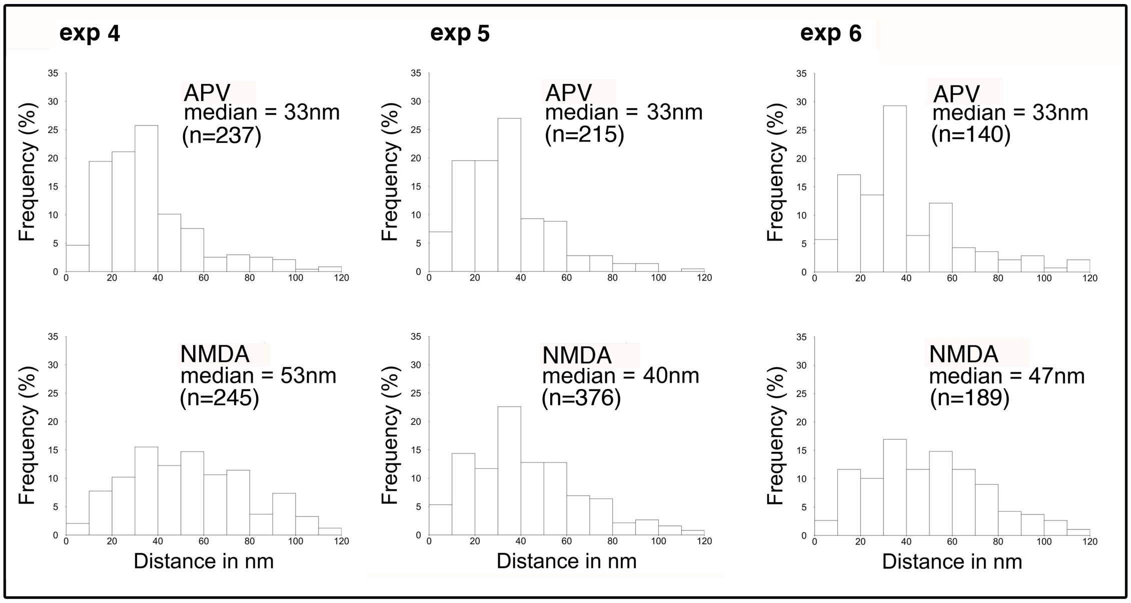

S2 Fig. Laminar distribution of IRSp53 label at the PSD under control/APV and excitatory conditions. Data for all six experiments is presented. Hippocampal cultures were exposed for 30 s to media containing high K^+ or NMDA. Distances of gold particles from the postsynaptic membrane were measured. Histograms show the percentage of label located in consecutive layers (10 nm bins). The median distance of gold particles from the postsynaptic membrane showed a significant increase under excitatory conditions in all six experiments ($P<0.0001$, Wilcoxon test, n=number of labels measured).
