# Supplementary material for: IRSp53 accumulates at the postsynaptic density under excitatory conditions
Source: PLoS One. 2017 Dec 28;12(12):e0190250. doi: 10.1371/journal.pone.0190250 (PMC5746258; doi:10.1371/journal.pone.0190250)
Supplement: S3 Fig — Data from all six experiments is presented. Labeling intensities in core and pallium compartments were estimated as: labelling intensity in PSD x percentage of label in each compartment. There was a significant difference in label intensity between basal (control and APV-treated) and stimulated (high K+ and NMDA-treated) samples in the pallium (P<0.0005, paired t test) but not in the core. (PDF) [file pone.0190250.s003.pdf]

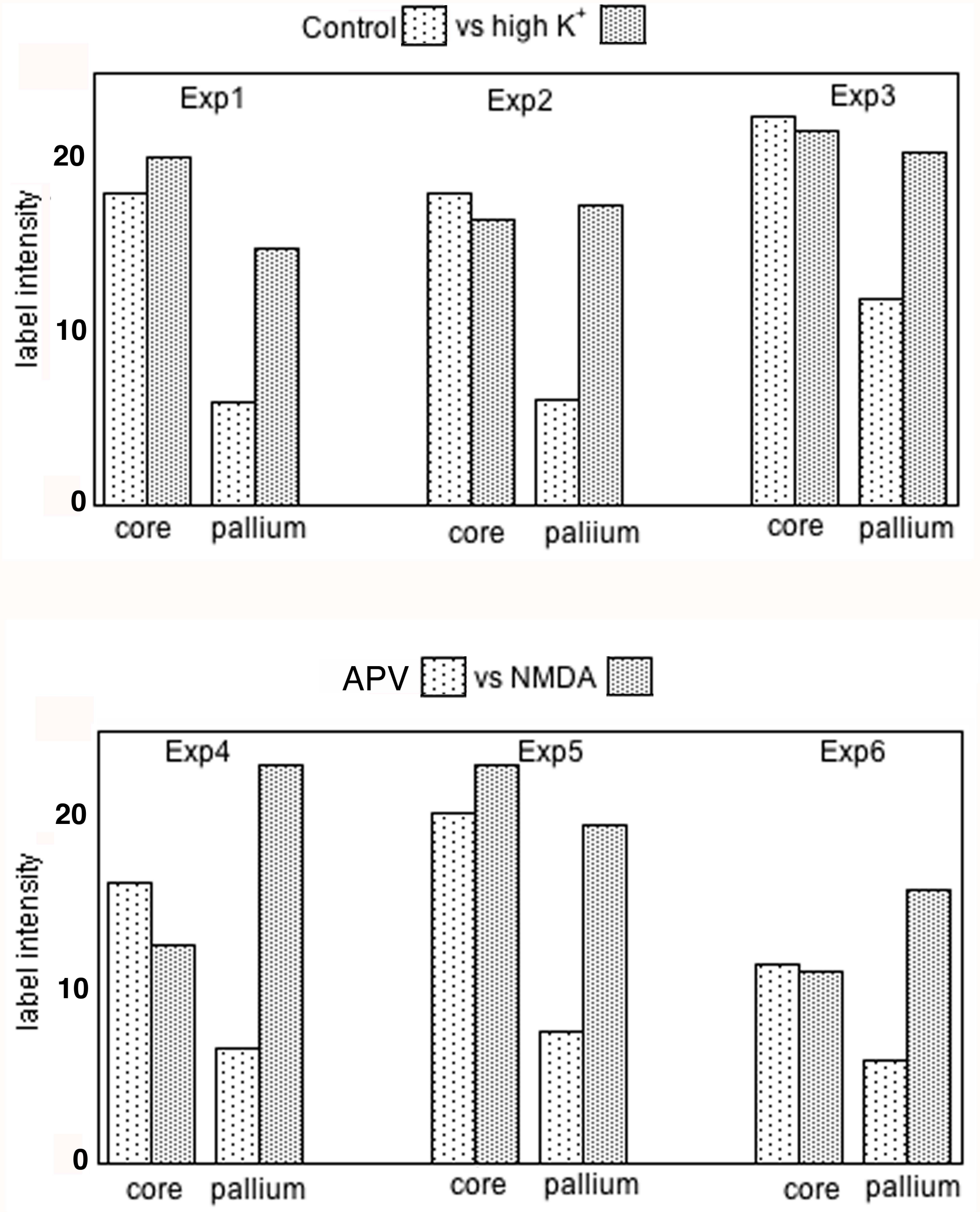

S3 Fig. Labelling intensity in the PSD core and PSD pallium under control and excitatory conditions. Data from all six experiments is presented. Labeling intensities in core and pallium compartments were estimated as: labelling intensity in PSD x percentage of label in each compartment. There was a significant difference in label intensity between basal (control and APV-treated) and stimulated (high K+ and NMDA-treated) samples in the pallium ($P<0.0005$, paired t test) but not in the core.
